# A Rapid and Visual Method for Nucleic Acid Detection of *Escherichia coli* O157:H7 Based on CRISPR/Cas12a-PMNT

**DOI:** 10.3390/foods12020236

**Published:** 2023-01-04

**Authors:** Wei Jiang, Chuan He, Lan Bai, Yifan Chen, Junwei Jia, Aihu Pan, Beibei Lv, Xueming Tang, Xiao Wu

**Affiliations:** 1 Biotechnology Research Institute, Shanghai Academy of Agricultural Sciences, Shanghai 201106, China; 2 Shanghai Key Laboratory of Agricultural Genetics and Breeding, Shanghai Academy of Agricultural Sciences, Shanghai 201106, China; 3 Crops Ecological Environment Security Inspection and Supervision Center (Shanghai), Ministry of Agriculture and Rural Affairs, Shanghai 201106, China; 4 School of Agriculture and Biology, Shanghai Jiao Tong University, Shanghai 200240, China

**Keywords:** foodborne pathogenic bacteria, *Escherichia coli* O157:H7, nucleic acid detection, POCT, visual method, CRISPR-Cas12a, PMNT

## Abstract

Rapid, accurate and visual point-of-care testing (POCT) methods for pathogenic bacteria detection are essential for avoiding foodborne diseases caused by pathogens or their toxins. In this study, we proposed a rapid and visual detection method that we named “Cas12aVIP”. By combining recombinase polymerase amplification (RPA), a CRISPR/Cas12a system and a cationic-conjugated polythiophene derivative (poly[3-(3′-N,N,N-triethylamino-1′-propyloxy)-4-methyl-2,5-thiophene hydrochloride] (PMNT) mixed with single-stranded DNA (ssDNA)), the solution turned red in the absence of the target DNA based on conformational modifications of the conjugated backbone of PMNT, whereas it displayed yellow, thus realizing the colorimetric detection of DNA. The Cas12aVIP method yielded high specificity and no interference from other nontargeted bacteria. The detection was accomplished in 40 min and the signal could be observed by the naked eye under natural light, presenting great potential for a variety of rapid nucleic acid detection applications without requiring technical expertise or ancillary equipment.

## 1. Introduction

Currently, the issue of food safety has attracted widespread attention around the world. Millions of people fall ill due to pathogenic bacteria infections each year, causing a severe economic burden [1]. Of the current detection methods, the conventional plate-cultures and colony-counting method is the gold standard, but this is a time-consuming and labor-intensive method [2]. Several nucleic acid-based assays, such as polymerase chain reaction (PCR), real-time PCR (RT-PCR), loop-mediated isothermal amplification (LAMP) and RPA with high sensitivity and specificity, have been extensively developed for foodborne pathogen detection [3,4]. However, these methods are not suitable for on-site and visual detection due to their reliance on equipment and reagents, and the requirement for professional personnel to operate them, limiting its popularization in resource-poor areas.

Recently, a CRISPR/Cas-based system and its combination with existing molecular biotechnology has achieved attomolar sensitivity in nucleic acid detection. This simple and flexible method with single-base resolution and on-site detection shows great application potential [5,6,7]. Through their combination with RPA technology, Cas12a and Cas13 were developed as DETECTR (DNA Endonuclease-Targeted CRISPR Trans Reporter), SHERLOCK (Specific High-sensitivity Enzymatic Reporter unLOCKing) and HOLMES (a one-HOur Low-cost Multipurpose highly Efficient System) systems for high sensitivity detection of nucleic acids [8,9,10]. Among them, the Cas12a-based system has shown a nonspecific endonuclease activity (trans-cleavage activity) against the surrounding ssDNA, which is an important mechanism in the application of CRISPR/Cas12a for nucleic acid detection. A recent study demonstrated that the CRISPR/Cas12a system coupled with RPA could be used to detect *Escherichia coli* O157:H7 [11]. However, blue light was required for visualization in the assay. Thus, a method that can output detection results under visible light without fluorescence detection instruments is necessary for applications in resource-poor settings.

Conjugated polymers have attracted the attention of academia and industry for their excellent optical properties of capturing light and amplification of fluorescence signals, which have made them a research hotspot in interdisciplinary research in biology, physics and chemistry [12,13,14]. Among them, PMNT has been extensively studied by scientists [15,16,17]. Polythiophene is easy to combine with macromolecules, such as DNA and protein, through electrostatic interactions that cause a steric configuration change of its conjugated backbone, leading to a change in its maximum absorption wavelength, so that the detection of macromolecules can be realized by the color changes in solution [18]. The main chain of PMNT takes a random coil and nonplanar configuration in water solution, and the solution is yellow (the maximum absorption wavelength is 397 nm). After adding ssDNA to the PMNT solution, it can form an interpolyelectrolyte complex with ssDNA (duplex) through electrostatic interactions, in which PMNT takes a highly conjugated and planar conformation. The solution then changed to red (the maximum absorption wavelength moved to 527 nm). The sensitive detection of nucleic acid can be realized through these color changes [19].

**Scheme 1:**
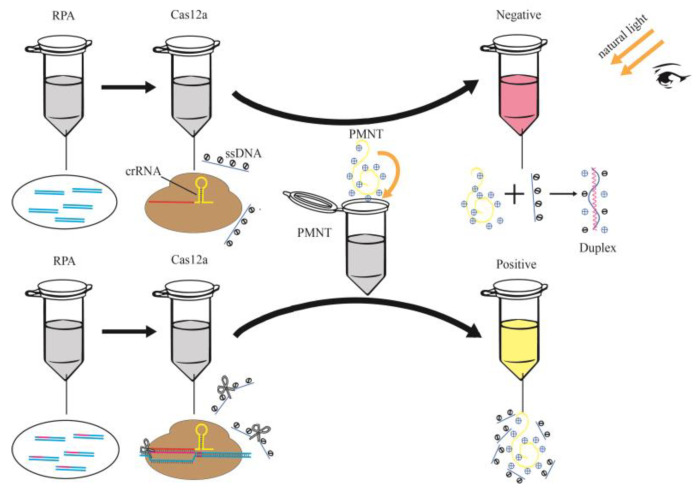
Schematic illustration of the colorimetric detection of DNA during the formation of PMNT/ssDNA duplex.

Here, we propose a rapid and visual detection method that we named Cas12a-based VIsual PMNT-involved (Cas12aVIP) detection through the combination of RPA, Cas12a and PMNT. The target double-stranded DNA (dsDNA) was amplified using RPA, followed by a reaction with Cas12a, crRNA and ssDNA. After cis-cleavage of the dsDNA with the Cas12a–crRNA complex, trans-cleavage activity of the complex was activated against ssDNA. When the cleaved ssDNA mixed with PMNT, the solution turned to red without target dsDNA, based on conformational modifications of the conjugated backbone of PMNT, whereas it displayed yellow with the existence of target dsDNA, realizing the colorimetric detection of DNA. The duration of the whole process was 40 min and the detection signal could be observed under natural light (Scheme 1).

## 2. Materials and Methods

### 2.1. Materials

All primers, ssDNA, CRISPR RNA (crRNA) and plasmid DNA of the foodborne microorganisms (*rfbE* gene in *E. coli* O157:H7 (GenBank: S83460.1), *toxRS* gene in *Vibrio parahaemolyticus* (GenBank: L11929.1), *Nuc* gene in *Staphylococcus aureus* (GenBank: V01281.1) and *Hly* gene in *Listeria monocytogenes* (GenBank: JF712529.1) [20,21,22,23] were synthesized by Sangon Biotech Ltd. (Shanghai, China). RNase inhibitor (40 U/µL) was purchased from Sangon Biotech Ltd. TwistAmp Basic kit for RPA assay was purchased from TwistDx Ltd. (Cambridge, UK). AxyPrep PCR Cleanup Kit for DNA purification was purchased from Corning Inc. (Corning, NY, USA). EnGen Lba Cas12a (Cpf1) kit for Cas12a-mediated target cleavage assay was purchased from New England Biolabs Inc. (Ipswich, MA, USA). PMNT was purchased from XHK Biochemicals Co., Ltd. (Changzhou, China). S1 endonuclease (100 U/µL), a ssDNA-specific enzyme, was purchased from Thermo Fisher Scientific Inc. (Waltham, MA, USA).

### 2.2. Chromogenic Reaction of PMNT after Binding to ssDNA of Various Sequence Length

ssDNA with different lengths of nucleotide sequences was designed and the color of the solution was observed by adding PMNT. The appropriate length of ssDNA was selected for subsequent experiments. The sequences are listed in Table S1. A total of 100 μM of PMNT and 1 μM of ssDNA was mixed with 1X NEBuffer r2.1 Reaction Buffer in a total volume of 30 μL.

### 2.3. Cas12a-Mediated Cleavage Assay

The crRNA was designed based on the PAM sequence on the *rfbE* gene in *E. coli* O157:H7 as follows: AAUUUCUACUGUUGUAGAUUUAGCGUUAGGUAUAUCGGAAGGA. Cas12a-mediated target cleavage assays were performed in 30 μL reaction volume using a EnGen Lba Cas12a (Cpf1) kit according to the optimized instruction manual. Each reaction contained 18 μL of nuclease-free water and substrate DNA, 1 μL of 40 U/μL RNase inhibitor, 3 μL of NEBuffer r2.1 Reaction Buffer (10×), 3 μL of 300 nM crRNA, 3 μL of 10 µM ssDNA and 1 µL of 1 µM EnGen Lba Cas12a (Cpf1). The reaction was performed at 37 °C for 10 min.

### 2.4. Feasibility Test

The reaction system contained 1 × NEBuffer r2.1 Reaction Buffer, 30 nM crRNA, 30 nM EnGen Lba Cas12a (Cpf1), 1.3 U/μL RNase inhibitor, 1 µM ssDNA, 30 nM positive/negative plasmid DNA, 100 µM PMNT and nuclease-free water. Eight different treatments were designed for the feasibility test: (1)–(4) were the same as the reaction system, but in the absence of (1) crRNA, (2) cas12a, (3) plasmid DNA or (4) ssDNA, and made up with nuclease-free water; (5) only contained nuclease-free water, buffer, ssDNA and PMNT; (6) was the same as the reaction system but with positive plasmid DNA; (7) was the same as the reaction system with negative plasmid DNA; (8) only contained nuclease-free water, S1 nuclease reaction buffer, S1 nuclease, ssDNA and PMNT. Three parallel reactions were performed for each treatment.

### 2.5. RPA Reaction Assay

Four pairs of highly specific primers for RPA reaction were synthesized according to sequences of *rfbE* gene in *E. coli* O157:H7, *toxRS* gene in *V. parahaemolyticus*, *Nuc* gene in *S. aureus* and *Hly* gene in *L. monocytogenes*, as described in previously reported work [24]. The sequences of the primers are presented in Table S2. RPA reactions were performed in a total volume of 50 μL using a TwistAmp Basic kit. Each reaction contained 29.5 μL of rehydration buffer, 2.5 μL of forward and reverse primers (10 μM), 13.8 μL of template and nuclease-free water and 2.5 μL of magnesium acetate (280 mM). The reaction was performed at 39 °C for 20 min. Following purification, the RPA amplicons were examined with agarose gel (2%) electrophoresis.

### 2.6. Detection of Bacterial DNA by Cas12aVIP

Plasmid DNA (3 pM) of *E. coli* O157:H7, *L. monocytogenes*, *S. aureus*, *V. parahaemolyticus* and an even mixture of all of them were RPA amplified. The RPA products of them were used as the substrate for Cas12a reaction. The crRNA used in this experiment corresponded to *E. coli* O157:H7. The substrate of DNA, Cas12a, crRNA and ssDNA was reacted at 39 °C for 10 min. PMNT was then added to the above reaction mixture and the results were observed under natural light. All detections were performed in triplicate.

## 3. Results

### 3.1. Color of PMNT after Binding to ssDNA of Various Sequence Length

**Figure 1:**
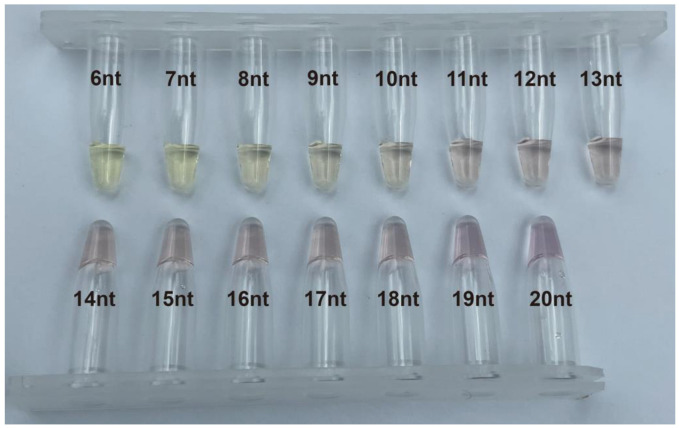
Color of mixed solution (PMNT with various lengths of ssDNA).

According to the color gradient shown in Figure 1, starting from ssDNA-10 nt, the mixed solution significantly changed from yellow to red under natural light in 1 min, indicating the formation of a PMNT/ssDNA complex. Since the solution color of ssDNA-15 nt was remarkably different from yellow, ssDNA-15 nt was selected for subsequent experiments.

### 3.2. Color Change of PMNT after Binding to ssDNA-15nt Cleaved by S1 Endonuclease

**Figure 2:**
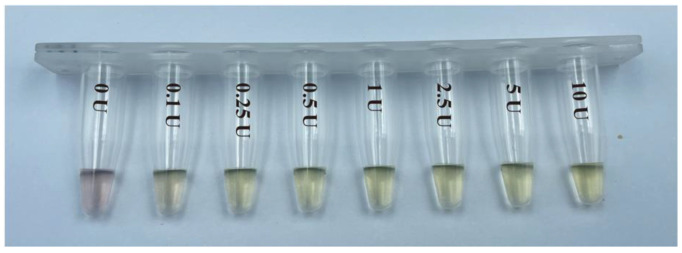
Color of mixed solution (PMNT with ssDNA-15 nt digested with various concentrations of S1 endonuclease).

A total of 1 μM of ssDNA-15 nt was reacted with S1 endonuclease at different concentrations (0, 0.1, 0.25, 0.5, 1, 2.5, 5 and 10 U) and then 100 μM of PMNT was added into the solution for chromogenic reaction. The reaction was performed in a total volume of 30 μL. The reaction solution displayed yellow with an ssDNA length of less than 10 nt, whereas above 10 nt it became red. As shown in Figure 2, the S1 endonuclease was able to degrade ssDNA-15 nt into short fragments at concentrations as low as 0.1 U, causing the color of the solution to be yellow, while it was red in the absence of S1 endonuclease.

### 3.3. Feasibility of Cas12a-PMNT-Based Visual Method for Nucleic Acid Detection

**Figure 3:**
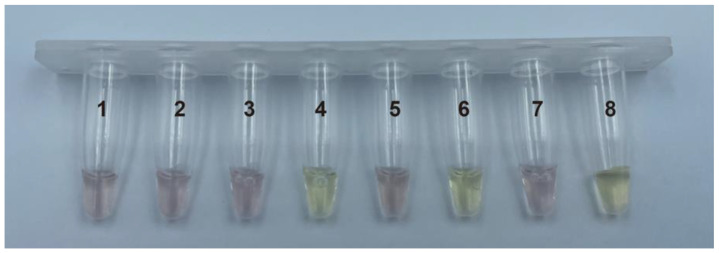
Feasibility of Cas12a–PMNT-based visual method for nucleic acid detection (1) NEBuffer + positive plasmid DNA + Cas12a + Rnase Inhibitor + ssDNA + PMNT; (2) NEBuffer + positive plasmid DNA + crRNA + Rnase Inhibitor + ssDNA + PMNT; (3) NEBuffer + crRNA + Cas12a + Rnase Inhibitor + ssDNA + PMNT; (4) NEBuffer + positive plasmid DNA + crRNA + Cas12a + Rnase Inhibitor + PMNT; (5) NEBuffer + ssDNA + PMNT; (6) NEBuffer + positive plasmid DNA + crRNA + Cas12a + Rnase Inhibitor + ssDNA + PMNT; (7) NEBuffer + negative plasmid DNA + crRNA + Cas12a + Rnase Inhibitor + ssDNA + PMNT; (8) S1 Reaction Buffer + S1 Nuclease + ssDNA + PMNT.

To explore the feasibility of the Cas12a–PMNT-based visual method for nucleic acid detection, eight experimental treatments were designed and the result is shown in Figure 3. Treatment (1)–(5) indicated that the red color of the solution was only related to the ssDNA. As a positive control, treatment (8) proved that the color of the solution would be yellow when ssDNA was cleaved. Treatment (6) and (7) showed that the method could detect whether the target or non-target DNA was contained in the solution. This result demonstrated that the method is feasible for the visual detection of nucleic acid samples.

### 3.4. Detection of Bacterial Samples

**Figure 4:**
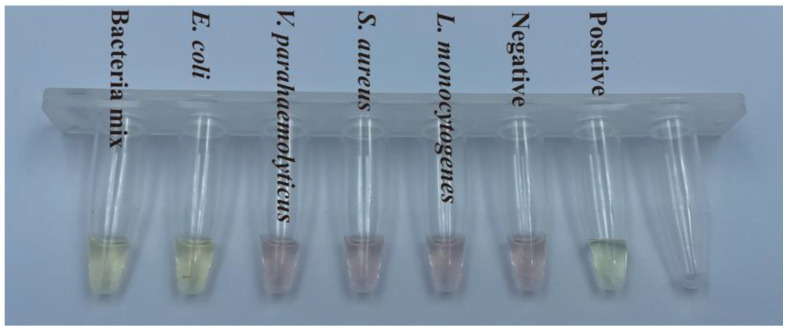
Detection result of bacterial samples.

Plasmid DNA of *E. coli* O157:H7, *L. monocytogenes*, *S. aureus*, *V. parahaemolyticus* and a mixture of all of them were RPA amplified. The crRNA used in this experiment corresponded to *E. coli* O157:H7. The RPA products of the four bacteria were added to the Cas12a reaction system. PMNT was added to the mixture after the reaction was completed. Since crRNA corresponded to *E. coli* O157:H7, ssDNA-15 nt were only degraded by Cas12a in the treatment containing the RPA product of *E. coli* O157:H7 and the mixture of all products, and the color of the solution was expected to be yellow. The solution color of the negative control was red in the absence of dsDNA, which was used as the substrate for the Cas12a reaction. As shown in Figure 4, the treatment with *V. parahaemolyticus*, *S. aureus* and *L. monocytogenes* did not contain the RPA product of *E. coli* O157:H7, so the solution was red, which fitted with the expected result. Only the treatment with the RPA product of *E. coli* O157:H7 and the mixture of all products showed a yellow color, which fitted with the expected color. Since the *rfbE* gene is specific for *E. coli* O157:H7, the above results showed that the Cas12aVIP method could detect *E. coli* O157:H7 specifically.

## 4. Discussion

In this study, we developed a rapid and visual DNA detection method by exploiting the isothermal and trace-DNA amplification feature of RPA, trans-cleavage activity of Cas12a, and chromatic phenomena of the PMNT/ssDNA complex under natural light. The PMNT/ssDNA complex turned red based on conformational modifications of the conjugated backbone of PMNT, whereas it displayed yellow with the existence of target dsDNA, realizing the colorimetric detection of DNA. Compared with regular detection using PCR, this method requires no precision instruments. Cas12aVIP can be accomplished in 40 min and the signal can be observed by the naked eye under natural light, realizing POCT of DNA. Since Cas12aVIP has high specificity, the *E. coli* O157:H7 samples could be detected accurately and the results would not have been affected by other foodborne microorganisms.

The molar weight of PMNT used in the experiment was much higher than that of ssDNA (by nearly 100 times). Subsequent experiments could be conducted to adjust the concentration of PMNT and the sequence length and concentration of ssDNA to balance the reliability of the detection with and economic factors. In addition, two uncapping operations were necessary to add Cas12a and PMNT after the RPA reaction, which increased the risk of aerosol contamination. In future follow-up studies, we will attempt to integrate three steps in a one-pot reaction by separating RPA reagents, the Cas12a enzyme and PMNT physically in one tube [25]. The detection could be accomplished in 30 min or less and uncapping contamination of samples could then be avoided. Furthermore, a photoactivatable CRISPR/Cas12a strategy [26] is also an option for improving one-pot nucleic acid detection.

## 5. Conclusions

We established a rapid, specific and visual nucleic acid detection method called Cas12aVIP based on Cas12a in combination with RPA and PMNT. Since Cas12aVIP is easily accessible for on-site nucleic acid detection under natural light, requiring no professionals, laboratory operation or fluorescence detection instruments, it can be used to reduce the workload of laboratories, presenting great potential for a variety of applications in the fields of food safety and environmental monitoring.

## Data Availability

Data is contained within the article or Supplementary Material.

## Supplementary Materials

The following supporting information can be downloaded at: https://www.mdpi.com/article/10.3390/foods12020236/s1, Table S1: Sequence of ssDNA used in this study; Table S2: Sequence of RPA primers used in this study.
