# Supplementary material for: A Rapid and Visual Method for Nucleic Acid Detection of Escherichia coli O157:H7 Based on CRISPR/Cas12a-PMNT
Source: Foods. 2023 Jan 4;12(2):236. doi: 10.3390/foods12020236 (PMC9858511; doi:10.3390/foods12020236)
Supplement: Supplementary file 1 [file foods-12-00236-s001.zip › foods-2088884-supplementary.pdf]

Supplementary Materials

A Rapid and Visual Method for Nucleic Acid Detection of *Escherichia coli* O157:H7 Based on CRISPR/Cas12a-PMNT

Wei Jiang [1,2,3,†], Chuan He [1,2,3,†], Lan Bai [1,2,3], Yifan Chen [1,2,3], Junwei Jia [1,3], Aihu Pan [2,3], Beibei Lv [1,2,3], Xueming Tang [1,2,3,4,*] and Xiao Wu [1,2,3,*]

[1] Biotechnology Research Institute, Shanghai Academy of Agricultural Sciences, Shanghai 201106, China
[2] Shanghai Key Laboratory of Agricultural Genetics and Breeding, Shanghai Academy of Agricultural Sciences, Shanghai 201106, China
[3] Crops Ecological Environment Security Inspection and Supervision Center (Shanghai), Ministry of Agriculture and Rural Affairs, Shanghai 201106, China
[4] School of Agriculture and Biology, Shanghai Jiao Tong University, Shanghai 200240, China
* Correspondence: xueming.tang@sjtu.edu.cn (X.T.); wuxiao@saas.sh.cn (X.W.)
† These authors contributed equally to this work.

Table S1. Sequence of ssDNA used in this study.

Name	Sequence (5' to 3')
ssDNA-6nt	TTTTTT
ssDNA-7nt	TTTTTTT
ssDNA-8nt	TTTTTTTT
ssDNA-9nt	TTTTTTTTT
ssDNA-10nt	TTTTTTTTTT
ssDNA-11nt	TTTTTTTTTTT
ssDNA-12nt	TTTTTTTTTTTT
ssDNA-13nt	TTTTTTTTTTTTT
ssDNA-15nt	TTTTTTTTTTTTTT
ssDNA-15nt	TTTTTTTTTTTTTTT
ssDNA-16nt	TTTTTTTTTTTTTTTT
ssDNA-17nt	TTTTTTTTTTTTTTTTT
ssDNA-18nt	TTTTTTTTTTTTTTTTTT
ssDNA-19nt	TTTTTTTTTTTTTTTTTTT
ssDNA-20nt	TTTTTTTTTTTTTTTTTTTT

Table S2. Sequence of RPA primers used in this study.

Primer Name	Sequence (5' to 3')	Amplicon Length (bp)
RPA-Ec-F	TCTTCATTTAGCTTT-GTTAGCGTTAGGTATATCGG	233
RPA-Ec-R	CCATATCACATGGATGTCCG-TATAAATGGACACAC	
RPA-Vp-F	ACTCGTATGAGAACGTGACATTGCGTATTT	426
RPA-Vp-R	TTGAACTCAGAAGGAGAAACAAGCAGATAG	
RPA-Sa-F	GCATCACAAACAGATAACGGCGTAAA-TAGAAG	139
RPA-Sa-R	ACATTAATTTAACCGTATCACCATCAATCGCT	
RPA-Lm-F	TTGATCACTCTGGAGGCTACGTTGCTCAATTC	158
RPA-Lm-R	GTTACCTGGCAAATAGATGGACGATGTGAAAT	
